# Orientation-Selective Retinal Circuits in Vertebrates

**DOI:** 10.3389/fncir.2018.00011

**Published:** 2018-02-07

**Authors:** Paride Antinucci, Robert Hindges

**Affiliations:** ^1^Centre for Developmental Neurobiology, King’s College London, London, United Kingdom; ^2^MRC Centre for Neurodevelopmental Disorders, King’s College London, London, United Kingdom

**Keywords:** orientation selectivity, retinal ganglion cell, amacrine cell, mouse, rabbit, primate, cat, zebrafish

## Abstract

Visual information is already processed in the retina before it is transmitted to higher visual centers in the brain. This includes the extraction of salient features from visual scenes, such as motion directionality or contrast, through neurons belonging to distinct neural circuits. Some retinal neurons are tuned to the orientation of elongated visual stimuli. Such ‘orientation-selective’ neurons are present in the retinae of most, if not all, vertebrate species analyzed to date, with species-specific differences in frequency and degree of tuning. In some cases, orientation-selective neurons have very stereotyped functional and morphological properties suggesting that they represent distinct cell types. In this review, we describe the retinal cell types underlying orientation selectivity found in various vertebrate species, and highlight their commonalities and differences. In addition, we discuss recent studies that revealed the cellular, synaptic and circuit mechanisms at the basis of retinal orientation selectivity. Finally, we outline the significance of these findings in shaping our current understanding of how this fundamental neural computation is implemented in the visual systems of vertebrates.

## Introduction

The retina is our window to the visual world. Visual scenes are highly processed by the retina before visual information, encoded in the coordinated firing of different types of retinal ganglion cells (RGCs), is transmitted to the brain through the optic nerve (Gollisch and Meister, 2010; Sanes and Masland, 2015). These different RGC types form functionally distinct ‘visual channels’ dedicated to the transmission of specific features present in the visual scene, such as directional motion, contrast, object size, or color. Recent studies that systematically classified RGCs according to their functional responses to visual stimuli and/or morphological properties indicate that there are around 20–30 of such visual channels (Helmstaedter et al., 2013; Robles et al., 2014; Baden et al., 2016; Bae et al., unpublished). Here, we will focus on one such channel in the retina, orientation selectivity.

**FIGURE 1:**
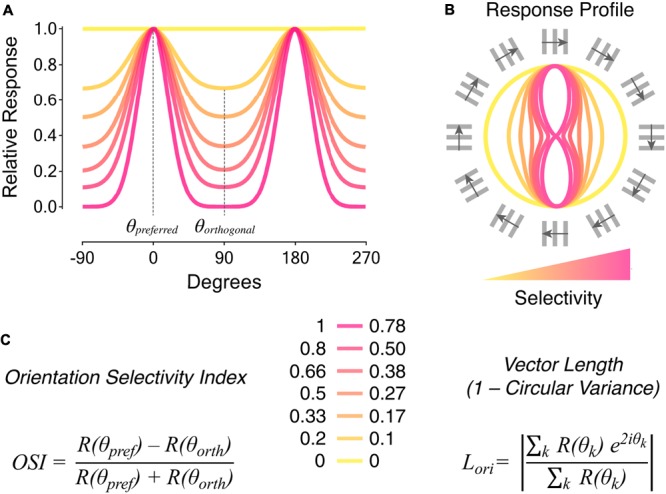
Metrics to quantify orientation selectivity in neural responses. **(A)** Tuning curves of neural responses to oriented visual stimuli. The color coding indicates different levels of orientation selectivity, from low (yellow) to high (magenta). The preferred orientation angle (*𝜃_preferred_*) corresponds to the angle of the stimulus eliciting maximal responses. The orthogonal orientation angle (*𝜃_orthogonal_*) corresponds to angles of stimuli oriented ± 90° relative to the preferred orientation angle. **(B)** Response profiles to oriented visual stimuli corresponding to the tuning curves represented in **(A)**. The orientation and direction of movement of square-wave gratings with 30° angular distance steps are indicated around. **(C)** Metrics typically used to quantify orientation selectivity in neural responses: orientation selectivity index (OSI; left), and vector length in orientation space (*L_ori_* also known as 1 – circular variance; right). Quantification of orientation selectivity for the responses in **(A,B)** is reported in the middle. Note that the two metrics have different sensitivities to tuned firing. The *OSI* consists in the difference between responses to preferred, *R(𝜃_pref_)*, and orthogonal, *R(𝜃_orth_)*, stimuli divided by their sum. On the other hand, *L_ori_* takes as input responses to all orientation angles, *R(𝜃_k_)*, in order to calculate the mean vector length in orientation space (*k* ranges from 0° to 180°). See Mazurek et al. (2014) for detailed descriptions and comparisons of the two metrics.

**FIGURE 2:**
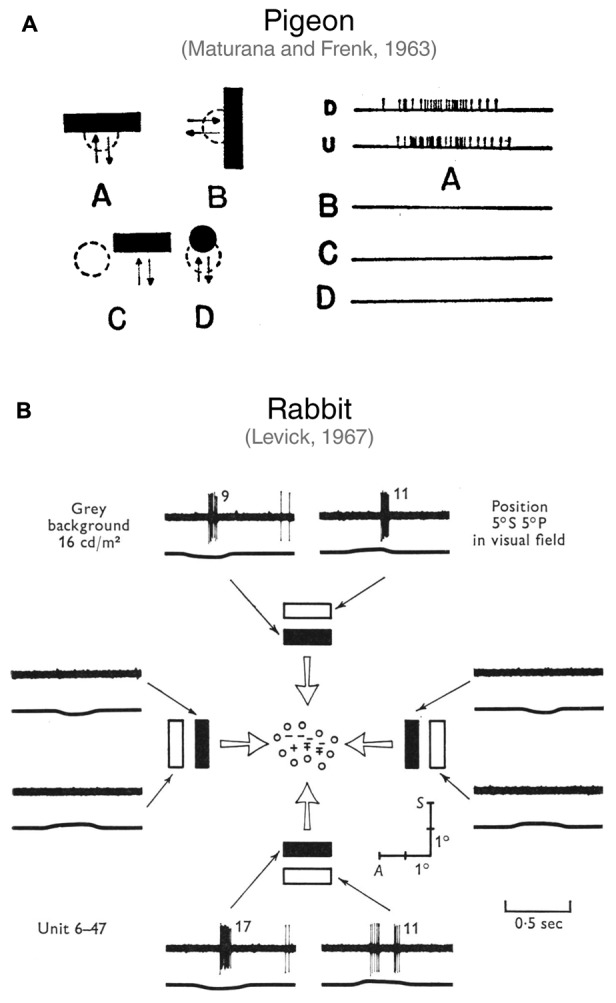
First studies describing orientation-selective ganglion cells in vertebrate retinae. **(A)** Discovery of horizontally tuned OSGCs in the pigeon retina by Maturana and Frenk (1963). In A (right side), the firing of a pigeon OSGC in response to a horizontal bar moving downward (D) or upward (U) is represented. As shown in B, C and D, the same cell does not respond to a vertically oriented bar moving leftward or rightward (B), nor to a horizontal bar presented over the receptive field surround (C), or to a small spot moving over the receptive field center (D). Image taken from Maturana and Frenk (1963) with permission. **(B)** Characterization of OSGCs in the rabbit retina by Levick (1967). Spiking responses of an OSGC to light or dark bars with different orientations moving across the receptive field center. The mapping of the receptive field center is also represented at the center of the schematic. The ‘+’ symbol indicates responses to a stationary spot at light ON; ‘–’, at light OFF; ‘ ± ’, at both light ON and OFF; ‘o’, no response detected. The traces show the spiking responses elicited by the bars (upper trace; number of spikes is reported after each response) and the output of a photomultiplier focused on the receptive field (lower trace; an upward deflection indicates light increase). Note that only horizontally oriented bars elicited responses. A, Anterior; S, superior. Image taken from Levick (1967) with permission.

Orientation selectivity was first discovered in cat primary visual cortex more than 50 years ago by Hubel and Wiesel (1962). They described it as the selectivity of neuronal firing for elongated visual stimuli oriented along a specific axis in the visual field (or *preferred orientation*), and suppression of firing when stimuli are oriented orthogonally to the preferred axis (or *orthogonal orientation*; see **Figure 1** for details on quantification of orientation selectivity). Shortly afterward, Maturana and Frenk (1963) and Levick (1967) identified orientation-selective ganglion cells (OSGCs) in the pigeon and rabbit retinae, respectively, suggesting that orientation-specific information is already evident in the output neurons of the retina (**Figure 2**). Since then, orientation-selective cells have been described in many vertebrate and invertebrate visual systems, including primates (Hubel and Wiesel, 1968), rodents (Niell and Stryker, 2008), fish (Nikolaou et al., 2012), and insects (Fisher et al., 2015). One of the reasons why orientation selectivity is so widely present across nervous systems and generated so early in visual processing likely lies in the fact that naturalistic visual scenes can be efficiently described by local, oriented filters with defined spatial structures (Olshausen and Field, 1996; Bell and Sejnowski, 1997; Simoncelli and Olshausen, 2001). The idea that orientation selectivity constitutes a fundamental feature of visual processing is reinforced by the fact that, as an artificial convolutional neural network learns an image classification task, the kernels/filters of its initial layer(s) typically become tuned to edge orientation (Krizhevsky et al., 2012; LeCun et al., 2015). Furthermore, face and object recognition in primates relies on the ability to identify, combine and relate oriented visual elements (Brincat and Connor, 2004; Dakin and Watt, 2009; Chang and Tsao, 2017).

Given the prominent role orientation selectivity plays in visual processing and perception, it is crucial to dissect how it emerges along the visual pathway, starting from the retina. Furthermore, comparing how this fundamental neural computation is implemented in different visual systems can provide us with important insights on how its underlying neural circuits could have evolved. In this review, we will start by reporting and comparing the orientation-selective cell types found in the retinae of various vertebrate species. We will then review the proposed mechanisms underlying retinal orientation selectivity at cellular and circuit levels. Finally, we will touch upon the contribution orientation selectivity generated within the retina might have to subsequent stages of visual processing occurring in higher brain areas.

## Orientation-Selective Cell Types in the Retina

After the initial discovery of orientation-selective cells in the retinae of pigeon (Maturana and Frenk, 1963) and rabbit (Levick, 1967), retinal orientation selectivity has since been reported in a multitude of other vertebrate species. These include macaque (Passaglia et al., 2002), cat (Levick and Thibos, 1980, 1982; Shou et al., 1995), mouse (Zhao et al., 2013; Chen et al., 2014; Pearson and Kerschensteiner, 2015; Baden et al., 2016; Nath and Schwartz, 2016, 2017), turtle (Sernagor and Grzywacz, 1995), goldfish (Damjanovic et al., 2009; Damjanovic et al., 2012; Johnston et al., 2014; Johnston and Lagnado, 2015), and zebrafish (Nikolaou et al., 2012; Antinucci et al., 2013, 2016b; Lowe et al., 2013). The study of orientation selectivity in the vertebrate retina has been pioneered in the rabbit, where (i) both orientation-selective amacrine cells (Bloomfield, 1991, 1994; Murphy-Baum and Taylor, 2015) and OSGCs (Levick, 1967; Amthor et al., 1989; Bloomfield, 1994; Venkataramani and Taylor, 2010, 2016) were initially found, (ii) the first pharmacological experiments were performed (Caldwell et al., 1978; Venkataramani and Taylor, 2010), and (iii) it was established that orientation and direction selectivity emerge through distinct mechanisms (He et al., 1998). In this section, we will describe the morphological and functional characteristics of OSGCs and orientation-selective amacrine cells from the various vertebrate species listed above (see **Table 1** for a summary).

**Table 1:** Summary of orientation-selective ganglion and amacrine cells in different vertebrate species.

Species	Study	Preferred orientation	Response polarity	Dendritic stratification	Dendritic elongation
**Orientation-selective ganglion cells**					
Pigeon	Maturana and Frenk, 1963	Horizontal			
Rabbit	Levick, 1967 (11%)	Horizontal, vertical	ON, OFF, ON-OFF		
	Caldwell et al., 1978	Horizontal, vertical	ON, OFF		
	Amthor et al., 1989	Vertical	OFF, ON-OFF	Bistratified	Not elongated
	Bloomfield, 1994	Horizontal, vertical	ON, OFF	Mono and bistratified	Not elongated
	Venkataramani and Taylor, 2010 (3%)	Horizontal, vertical	OFF	Monostratified?	Not elongated
	Venkataramani and Taylor, 2016 (2%)	Horizontal	ON	Monostratified	Elongated
Cat (*Orientation bias*)	Levick and Thibos, 1980, 1982; (70%) Thibos and Levick, 1985	Horizontal, vertical, oblique	ON, OFF		
	Shou et al., 1995 (61%)	Horizontal, vertical, oblique	ON, OFF		
Macaque	Passaglia et al., 2002	Horizontal, vertical, oblique	ON, OFF		
Mouse	Baden et al., 2016 (14.5%)	Horizontal	ON, OFF, ON-OFF	Mono (OFF) and bistratified (ON, ON-OFF)	Not elongated
		Vertical	ON, OFF, ON-OFF	Mono (OFF) and bistratified (ON, ON-OFF)	Not elongated
		Oblique	ON	Bistratified	Not elongated
	Nath and Schwartz, 2016	Horizontal	ON	Bistratified	Elongated
		Vertical	ON	Bistratified	Not elongated
	Nath and Schwartz, 2017	Horizontal	OFF	Monostratified	Not elongated
		Vertical	OFF	Monostratified	Not elongated
Goldfish	Damjanovic et al., 2009	Horizontal, vertical	ON-OFF		
Zebrafish	Nikolaou et al., 2012; Lowe et al., 2013 (10%)	Horizontal, vertical, oblique			
	Antinucci et al., 2016b (10%)	Horizontal, vertical, oblique	OFF, ON-OFF	Mono and bistratified	
**Orientation-selective amacrine cells**					
Rabbit	Bloomfield, 1991, 1994	Horizontal, vertical	ON, OFF	Monostratified	Not elongated
		Horizontal, vertical	ON, OFF	Monostratified	Elongated
	Murphy-Baum and Taylor, 2015	Horizontal	ON	Bistratified (displaced polyaxonal)	Elongated
Mouse	Nath and Schwartz, 2017	Vertical	OFF	Monostratified	Elongated
Zebrafish	Antinucci et al., 2016b	Horizontal, vertical, oblique	OFF, ON-OFF	Mono and bistratified	Elongated

*The functional and morphological features of orientation-selective cells are reported together with the relative study. Studies are grouped according to species, research group and cell types under investigation. When explicitly stated by the authors, the fraction of orientation-selective ganglion cells found (or estimated) among the whole population of ganglion cells is reported in brackets next to the study.*

### Orientation-Selective Ganglion Cells

**FIGURE 3:**
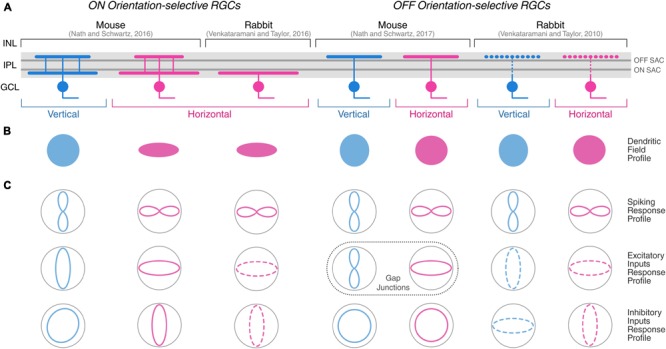
Morphological and physiological features of orientation-selective retinal ganglion cells in mouse and rabbit. Schematic summarizing the morphological **(A,B)** and physiological **(C)** properties of ON and OFF OSGCs in mouse (Nath and Schwartz, 2016, 2017) and rabbit (Venkataramani and Taylor, 2010, 2016). Dendritic stratification **(A)** in the inner plexiform layer (IPL), and planar dendritic field profiles **(B)** of OSGCs are displayed. Dark gray lines in the IPL indicate ON and OFF choline acetyltransferase (ChAT)-labeled strata corresponding to ON and OFF starburst amacrine cell (SAC) neurites, respectively. The IPL dendritic stratification of rabbit OFF OSGCs is represented with dashed lines because it was not explicitly described by Venkataramani and Taylor (2010), but other studies have reported both mono- and bistratified OFF OSGCs in rabbit (see **Table 1**). **(C)** Response profiles of OSGC spiking (top), excitatory inputs (middle), and inhibitory inputs (bottom). Dashed lines of excitatory and inhibitory inputs response profiles in rabbit OSGCs indicate estimated profiles from responses recorded only during preferred and orthogonal orientation stimulation. In mouse OFF OSGCs, the tuned response profiles of gap junction-mediated electrical inputs are indicated (dashed box) instead of excitatory inputs from chemical synapses. INL, inner nuclear layer; GCL, ganglion cell layer. Diagram modified and expanded from Antinucci et al. (2016a) with permission.

The vertebrate species in which OSGCs have been best characterized are rabbit and mouse (see **Figure 3** for the most comprehensively described OSGC types). In rabbit, only OSGC types tuned to stimuli oriented along the cardinal axes of the visual field (i.e., horizontal and vertical) have been found (Levick, 1967; Caldwell et al., 1978; Amthor et al., 1989; Bloomfield, 1994; Venkataramani and Taylor, 2010, 2016). On the other hand, cells tuned to cardinal (Nath and Schwartz, 2016, 2017) as well as oblique orientations (Baden et al., 2016) have been identified in mouse. In general, it appears that among the vertebrate species where OSGCs have been described, cells tuned to cardinal orientations are always present, whereas OSGCs tuned to oblique orientations have been reported only in mouse (Baden et al., 2016), cat (Levick and Thibos, 1980, 1982; Thibos and Levick, 1985; Shou et al., 1995), macaque (Passaglia et al., 2002), and zebrafish (Nikolaou et al., 2012; Lowe et al., 2013; Antinucci et al., 2016b).

In regard to the response polarity of OSGCs, ON (i.e., responses to light onset), OFF (i.e., responses to light offset), and ON-OFF (i.e., responses to both light onset and offset) cell types were found across vertebrates. In rabbit, most studies seem to show that the response polarity of OSGCs is either ON or OFF (Caldwell et al., 1978; Bloomfield, 1994; Venkataramani and Taylor, 2010, 2016). However, Levick (1967) and Amthor et al. (1989) also reported a few OSGCs with an ON-OFF receptive field center (see **Figure 2B**, for example). In mouse, OSGCs with all types of response polarity have been identified (Baden et al., 2016; Nath and Schwartz, 2016, 2017). ON-OFF OSGCs typically possess a bistratified dendritic arbor across vertebrate species (Amthor et al., 1989; Antinucci et al., 2016b; Baden et al., 2016). Yet, OSGCs with ON or OFF receptive field centers do not always show monostratified dendritic arbors residing in the corresponding lamina of the inner plexiform layer (IPL). For example, in mouse, the dendrites of ON OSGCs consistently stratify in two IPL laminae (Baden et al., 2016; Nath and Schwartz, 2016), one directly above OFF starburst amacrine cell (SAC) neurites and the other just below the ON SAC lamina (**Figure 3A**, left). Similarly, OFF OSGCs with bistratified dendritic arbors were also found, but only in the rabbit retina (Amthor et al., 1989; Bloomfield, 1994). The planar dendritic morphology of OSGCs has been thoroughly analyzed only in rabbit and mouse (**Figure 3B**). For most cell types characterized, the planar shape of OSGC dendritic arborisations does not appear to be elongated (Amthor et al., 1989; Bloomfield, 1994; Venkataramani and Taylor, 2010; Baden et al., 2016; Nath and Schwartz, 2016, 2017). However, ON horizontal OSGCs in both species are an exception (Nath and Schwartz, 2016; Venkataramani and Taylor, 2016). Their dendritic arbors are highly elongated along the horizontal axis of the retina (**Figure 3B**). Interestingly, the orientation of the dendritic arbor elongation coincides with their preferred stimulus orientation (i.e., horizontally oriented; **Figure 3C**). This morphological feature of ON horizontal OSGCs, that appears to be conserved in two different vertebrate species, could potentially contribute to their selective firing (see below for detailed discussions on the mechanisms underlying the orientation tuning of this OSGC type). A link between the orientation bias found among most cat RGCs and the elongation of their dendritic fields and receptive field centers has also been suggested (Hammond, 1974; Levick and Thibos, 1980, 1982; Leventhal and Schall, 1983) (see below for a more detailed discussion).

### Orientation-Selective Amacrine Cells

**FIGURE 4:**
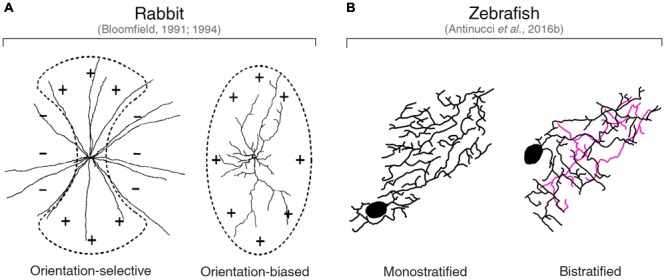
Orientation-tuned amacrine cells in rabbit and zebrafish. **(A)** Planar dendritic morphology of the two classes of orientation-tuned amacrine cells found in the rabbit retina by Bloomfield (1991, 1994). ‘Orientation-selective’ amacrine cells have a circular dendritic field (left). ‘Orientation-biased’ amacrine cells are characterized by a highly elongated dendritic field (right). Schematic representations of the corresponding receptive fields are overlaid on top of the dendritic arbors. ‘+’ and ‘–’ symbols indicate excitatory and inhibitory inputs, respectively. Images taken from Bloomfield (1994) with permission. **(B)** Planar dendritic morphology of the two types of orientation-tuned amacrine cells found in the larval zebrafish retina by Antinucci et al. (2016b). Note the high degree of dendritic elongation similar to that observed in rabbit ‘orientation-biased’ amacrine cells. The color coding of neurites indicates the different IPL laminae they are located. Black and magenta lines indicate neurites in OFF and ON laminae, respectively. Images taken from Antinucci et al. (2016b) with permission.

The only vertebrate species in which orientation-selective amacrine cell types have been described are rabbit (Bloomfield, 1991, 1994; Murphy-Baum and Taylor, 2015), zebrafish (Antinucci et al., 2016b), and mouse (Nath and Schwartz, 2017). In the rabbit retina, three orientation-selective amacrine cell classes have been found. Bloomfield (1991, 1994) has characterized two classes of amacrine cells showing tuning for cardinal orientations. One group comprises wide-field amacrine cells with long, radially extending neurites that were termed ‘orientation-selective,’ since orientation tuning appears to arise through an asymmetric inhibitory mechanism (**Figure 4A**, left). The second group consists of medium-field amacrine cells with highly elongated dendritic arbors that were classified as ‘orientation-biased’ (**Figure 4A**, right). The preferred orientation of the latter cells coincides with the orientation of their dendritic field and they do not seem to receive asymmetric inhibitory inputs (see below for a detailed discussion on the mechanisms underlying orientation tuning of this amacrine cell type). For both ‘orientation-selective’ and ‘orientation-biased’ amacrine cell classes, Bloomfield (1991, 1994) reported ON as well as OFF types, which all have monostratified dendritic arbors extending along the central lamina of the IPL.

Murphy-Baum and Taylor (2015) characterized a third class of orientation-tuned amacrine cells in the rabbit retina. This class consists of a well-defined type of polyaxonal, wide-field amacrine cells with ON response polarity and cell bodies displaced in the ganglion cell layer. They are consistently tuned to horizontally oriented visual stimuli and, like the ‘orientation-biased’ amacrine cells described by Bloomfield (1991, 1994), their dendritic arbor is highly elongated along the preferred orientation axis (i.e., the major axis of their dendritic field extends horizontally). However, unlike ‘orientation-biased’ amacrine cells, these polyaxonal amacrine cells show a bistratified arrangement of their neurites in the IPL. Specifically, their dendrites stratify just above the OFF SAC lamina, as well as in the IPL lamina between ON and OFF SAC neurites, whereas their axons narrowly extend just above the ON SAC lamina.

Antinucci et al. (2016b) found two types of orientation-tuned amacrine cells in the larval zebrafish retina (**Figure 4B**). These amacrine cell types are characterized by highly elongated dendritic fields with orientations that match their preferred stimulus orientations. Cardinal and oblique orientation preferences are represented in both types. However, they differ in terms of their dendritic stratification pattern in the IPL: one type has a monostratified dendritic arbor extending in the OFF lamina just below the inner nuclear layer border (**Figure 4B**, left), whereas the other type shows a bistratified dendritic arbor with an additional branching in the ON portion of the IPL (**Figure 4B**, right). The high planar elongation of their dendritic arbors is homologous to that found in rabbit ‘orientation-biased’ (Bloomfield, 1991, 1994) and polyaxonal amacrine cells (Murphy-Baum and Taylor, 2015), indicating a conserved morphological property of orientation-tuned amacrine cells. Recently, this morphological feature has also been found in some mouse OFF orientation-selective amacrine cells with monostratified dendritic trees (Nath and Schwartz, 2017).

## Mechanisms Underlying Retinal Orientation Selectivity

What are the neural bases underlying orientation selectivity in the cell types described above? In studies that first reported orientation selectivity in RGCs (Maturana and Frenk, 1963; Levick, 1967), it was proposed that this visual property likely emerges from an asymmetric interaction between excitatory and inhibitory inputs converging onto RGCs, given the strong suppression of firing during stimulation with the non-preferred, orthogonal orientation (**Figure 2**). Subsequent studies that revealed the presence of orientation-selective amacrine cells indicated possible origins of this differential firing (Bloomfield, 1991, 1994; Murphy-Baum and Taylor, 2015; Antinucci et al., 2016b; Nath and Schwartz, 2017). In this section, we will explore the various morphological and synaptic mechanisms reported to contribute to orientation selectivity in RGCs, amacrine and bipolar cells. In some species, there has been sufficient progress to start outlining potential wiring diagrams of orientation-selective retinal circuits, which take us closer to answering the question: Where does orientation selectivity first emerge in the retina?

### Morphological Mechanisms

In the quest of finding the neural origins of orientation selectivity in the retina, several studies have tried to find links between morphological features and selective firing in response to elongated visual stimuli. The morphology of the dendritic arbor, for example, has been shown to affect the selectivity for oriented stimuli in various cell types and vertebrate species. As pointed out above, in some orientation-selective cells with elongated dendritic arbors, there is a strong correlation between the orientation of the dendritic field and the preferred stimulus orientation. Key examples are the orientation-tuned amacrine cell types with highly elongated dendritic arbors found in rabbit, mouse and zebrafish retinae (**Figure 4**) (Bloomfield, 1991, 1994; Murphy-Baum and Taylor, 2015; Antinucci et al., 2016b; Nath and Schwartz, 2017).

Bloomfield (1991, 1994) reported a population of amacrine cells in the rabbit retina with highly elongated dendritic fields oriented along the horizontal and vertical axes of the retina. As mentioned before, he defined these cells as ‘orientation-biased’ since their firing selectivity seemed to arise exclusively from the excitatory inputs received along the major axis of their dendritic arbor (**Figure 4A**, right). During orthogonal orientation stimulation, these cells still show spiking responses, albeit with lower amplitude than responses to the preferred orientation. In addition, no inhibitory currents were observed during preferred or orthogonal orientation stimulations, indicating the absence of a mechanism involving an antagonistic surround. Together with the fact that these amacrine cells’ elongated receptive and dendritic fields have the same orientation and are comparable in size, these findings strongly suggest that the architecture of their dendritic arbors provide the structural basis for their elongated receptive fields. This in turn underlies their preference for oriented stimuli that maximally cover the center receptive field area. Essentially, the orientation selectivity of these cells can be explained on anatomical grounds alone without the need of any interplay between excitatory and inhibitory inputs.

Another example of orientation-selective amacrine cells whose firing selectivity can be explained by morphological properties of the dendritic arbor are the polyaxonal, wide-field amacrine cells identified by Murphy-Baum and Taylor (2015) in rabbit. These displaced ON-type amacrine cells have bistratified dendritic arbors that exhibit a consistent elongation along the horizontal axis of the retina, which in the rabbit retina coincides with the orientation of the visual streak (i.e., the retinal area with the highest visual acuity). The shape of their receptive field closely matches the spatial extent and orientation of their dendritic field. Moreover, stimulation with light bars showed that the orientation tuning of both their spiking output and excitatory currents strongly correlates with the orientation of their dendritic field elongation axis. Inhibitory currents, on the other side, are weakly tuned to the preferred orientation and therefore do not enhance the tuning of their spiking output. Like the cells described by Bloomfield (1991, 1994), the orientation selectivity of these polyaxonal amacrine cells is primarily due to the arrangement of excitatory inputs onto their elongated dendritic arbor. It is worth mentioning that, in addition to this morphological mechanism, the responses of these cells also depend on the stimulus structure (i.e., its spatial frequency). Briefly, a quasi-linear summation of contrast in the receptive field center, which is mediated by modulation of tonic ON excitatory inputs and crossover OFF inhibitory inputs, sharpens their orientation selectivity by differentially suppressing responses to orthogonal versus preferred orientation gratings according to the stimulus spatial frequency. This results in suppression of responses to high spatial frequencies and increased sensitivity to stimuli with a spatial frequency matching the width of their receptive field minor axis (Murphy-Baum and Taylor, 2015).

In the larval zebrafish retina, orientation-selective amacrine cell types with highly elongated dendritic fields were described by Antinucci et al. (2016b). These amacrine cells release gamma-aminobutyric acid (GABA) and comprise two types that possess either monostratified or bistratified dendritic arbors (**Figure 4B**). Calcium imaging of these cells in the intact retina followed by structural analyses of their morphology revealed that their preferred stimulus orientation coincides with the orientation of their dendritic field major axis. Electrophysiological recordings to isolate synaptic inputs were not performed in these cells. However, the fact that the degree of orientation tuning (i.e., their orientation selectivity index, OSI, **Figure 1C**) is directly proportional to the magnitude of their dendritic arbor elongation (i.e., the more elongated the dendrites, the higher the OSI), suggests that their response profile likely depends on how much a given stimulus is capable of exciting their receptive field center. In addition to cardinal orientations preference, like the rabbit amacrine cells described above, some of these cells are also selective to obliquely oriented stimuli. This is a feature that, among the few orientation-selective amacrine cell types described in vertebrates to date, has been observed only in the zebrafish retina and is in line with the presence of OSGCs tuned to oblique stimuli in the larval zebrafish retina (Lowe et al., 2013; Antinucci et al., 2016b).

In mouse, the OFF orientation-selective amacrine cells recently found by Nath and Schwartz (2017) have highly elongated, vertically oriented dendritic arbors. Like in rabbit and zebrafish, their orientation preference coincides with the orientation of their dendritic tree. Crucially, they co-stratify their dendrites with those of OFF vertically tuned OSGCs in the IPL lamina just above the OFF SAC lamina. In addition, Nath and Schwartz (2017) showed that these amacrine cells are electrically coupled to OFF vertically tuned OSGCs through connexin 36-mediated gap junctions, which makes them primary candidates for imparting orientation selectivity to postsynaptic OFF OSGCs.

Is dendritic morphology a key feature for orientation selectivity also in RGCs? Among rabbit and mouse OSGCs, only ON horizontally selective OSGCs have been reported to display an asymmetric, elongated dendritic arbor (Nath and Schwartz, 2016; Venkataramani and Taylor, 2016). In particular, their dendritic field is oriented along the horizontal axis of the retina and coincides with their preferred stimulus orientation (**Figure 3B**). This property is unlikely the only factor contributing to their orientation tuning since these cells also receive stereotyped and highly tuned excitatory and inhibitory inputs (**Figure 3C**, see below). Strikingly, the extent to which dendritic morphology contributes to their orientation selectivity differs in the two species. In rabbit, blocking GABAergic inputs completely abolishes orientation-selective spiking (see below), strongly suggesting that inhibition is the key mechanism underlying their tuning (Venkataramani and Taylor, 2016). However, in mouse, dendritic structure appears to have an important function since pharmacologically blocking all inhibitory inputs received by these cells does not affect the tuning of their firing or their excitatory currents. Thus, their orientation tuning presumably arises from the asymmetric integration of excitatory inputs as a consequence of their elongated dendritic arbor (Nath and Schwartz, 2016).

In the cat retina, the orientation bias found among the vast majority (∼60–70%) of RGCs (Levick and Thibos, 1980, 1982; Thibos and Levick, 1985) appears to be linked to dendritic field elongation as well as location within the retina (Leventhal and Schall, 1983). Specifically, the distributions of preferred stimulus orientation and dendritic arbor elongation across the retina seem to match, namely in both cases the major axis of both the preferred stimulus and dendritic field is oriented radially with respect to the *area centralis* of the retina. Following this observation, together with the report that most cat RGCs have an elliptical receptive field center (Hammond, 1974), it has been suggested that the sensitivity of cat RGCs to orientation is likely a consequence of their elongated dendritic fields (Leventhal and Schall, 1983; Peichl and Wassle, 1983).

Among the various factors that determine the selective firing of retinal cells, morphological features are probably the hardest in which a causal link to specific functional properties can be experimentally established. This is mainly because we still have a limited understanding of how dendritic structures are shaped and, consequently, lack tools to manipulate the morphology of individual cell types in a targeted way. The findings presented above show correlational observations between dendritic arbor elongation and orientation tuning. In most cases, the correlations are strong and consistent with physiological data. However, only experiments designed to selectively alter dendritic field morphology and directly observe the functional consequences in neuronal firing will be able to determine whether morphology is the primary cause of orientation-selective responses in the cell types described above.

### Synaptic Mechanisms

We have seen that morphological features are likely at the basis of orientation selectivity in certain retinal cell types and that cell morphology appears to have a stronger role in some vertebrate species than in others. In cell types where morphology is not a key contributor to selective firing, orientation tuning probably results from the integration of asymmetric synaptic inputs from amacrine and bipolar cells. As mentioned above, the first hints that asymmetric inputs play a crucial role in generating orientation-selective firing came from early studies that revealed the presence of OSGCs in the retina (Maturana and Frenk, 1963; Levick, 1967). Levick (1967), for example, observed that when rabbit OSGCs are stimulated with orthogonally oriented bars of progressively longer length, their firing stops when the bar length reaches a certain critical value. He interpreted this result as an inhibitory effect from an asymmetric receptive field surround and suggested that along the axis of selectivity the antagonistic surround of OSGCs is effectively incomplete. Analogous results, again in rabbit OSGCs, were also reported by Bloomfield (1994) who observed a strong membrane potential hyperpolarisation in response to orthogonally oriented bars that was directly proportional to stimulus length.

Evidence that inhibition from amacrine cells is necessary for orientation selectivity in rabbit OSGCs came from pharmacological experiments in which different inhibitory receptors where selectively blocked (see **Table 2** for a summary). Caldwell et al. (1978) found that blocking GABA_A_ receptors through picrotoxin completely abolishes orientation-selective firing in both ON and OFF-type OSGCs, whereas using strychnine to block glycine receptors did not produce such an effect. These initial observations were further corroborated through a series of elegant studies carried out by Venkataramani and Taylor (2010, 2016), who demonstrated that GABAergic amacrine cells act through GABA_A_ receptor-mediated mechanisms to generate the orientation-selective firing of rabbit OSGCs. This research group systematically recorded the effects of various pharmacological manipulations on excitatory and inhibitory inputs received by different types of rabbit OSGCs, specifically ON horizontal, OFF horizontal and OFF vertical OSGCs. Interestingly, they found not only that GABA_A_ receptor blockade causes different effects depending on the OSGC type under study, but also revealed that glycinergic amacrine cells as well contribute to the tuning of some OSGCs (see **Table 2** and below).

**Table 2:** Summary of pharmacological experiments in vertebrate orientation-selective ganglion cells.

Species	Study	Cell type	Receptors blocked (drug used)	Effects on orientation-selective Ganglion cells
**Pharmacological experiments**				
Rabbit	Caldwell et al., 1978	ON, OFF	GABA-A (Picrotoxin)	Abolishment of orientation-selective firing
			Glycine (Strychnine)	No effect on orientation-selective firing
	Venkataramani and Taylor, 2010	OFF Horizontal	GABA-A (Gabazine)	Abolishment of inhibitory currents during orthogonal stimulation; Almost complete loss of orientation selectivity in excitatory currents
			Glycine (Strychnine)	Reduction in inhibitory currents during orthogonal stimulation; Decrease in orientation selectivity of excitatory currents
		OFF Vertical	GABA-A (Gabazine)	Increased disinhibition during orthogonal stimulation; Partial loss of orientation selectivity in excitatory currents
			Glycine (Strychnine)	Abolishment of disinhibition during preferred stimulation; Negligible effect on orientation selectivity of excitatory currents
	Venkataramani and Taylor, 2016	ON Horizontal	GABA-A (Gabazine)	Abolishment of orientation-selective firing; Increase in inhibitory currents during preferred stimulation; Increase in excitatory currents during orthogonal stimulation
Mouse	Nath and Schwartz, 2016	ON Horizontal and Vertical	GABA-A (Gabazine)	Reduction in inhibition but no effect on orientation selectivity
			Glycine (Strychnine)	Reduction in inhibition but no effect on orientation selectivity
			GABA-A and Glycine (Gabazine + Strychnine)	Complete suppression of inhibitory currents; No effect on orientation selectivity of excitatory currents
	Nath and Schwartz, 2017	OFF Horizontal and Vertical	Gap Junctions (Meclofenamic acid; bath)	Abolishment of firing; Decrease in orientation selectivity of subthreshold voltage responses
			Gap Junctions (Quinine; intracellular)	Reduction in firing; Decrease in orientation selectivity of subthreshold voltage responses
			GABA-A (Gabazine)	Reduction in inhibitory currents
			Glycine (Strychnine)	Reduction in inhibitory currents
			GABA-A and Glycine (Gabazine + Strychnine)	Complete suppression of inhibitory currents
Zebrafish	Antinucci et al., 2016b	Horizontal, vertical, oblique	GABA-A (Picrotoxin)	Decrease in orientation-selective calcium responses
			Glycine (Strychnine)	No effect on orientation-selective calcium responses

*The observed effects are reported together with the relative study. Studies are grouped according to species, research group and cell types under investigation*.

A homologous role of GABAergic inhibition through amacrine cells has been found in the larval zebrafish retina (Antinucci et al., 2016b). Pharmacologically blocking GABA_A_ receptors using picrotoxin dramatically reduced the fraction of orientation-selective responses recorded from RGCs through calcium imaging. In this study, the authors were also able to optogenetically ablate genetically defined GABAergic amacrine cells that show orientation tuning (**Figure 4B** and description above) and examine the functional consequences. Strikingly, the complete ablation of this class of orientation-selective amacrine cells produced an impairment in OSGC responses equivalent to the one produced by blocking GABAergic inhibition. Importantly, this result in zebrafish provides the first direct indication that inhibitory orientation-selective amacrine cells are crucial circuit elements necessary to generate orientation tuning in RGCs. The current evidence in rabbit points in the same direction and future experiments will likely elucidate to what extent orientation-selective amacrine cells play the same role in the rabbit retina.

If OSGCs indeed receive inhibitory inputs from orientation-selective amacrine cells, this should be evident in the tuning of synaptic currents recorded from their soma during visual stimulation. Recent studies in rabbit and mouse performed whole-cell voltage clamp recordings in OSGCs to isolate their excitatory and inhibitory currents (Venkataramani and Taylor, 2010; Antinucci et al., 2016a; Nath and Schwartz, 2016; Venkataramani and Taylor, 2016; Nath and Schwartz, 2017). Strikingly, both rabbit and mouse OSGCs receive synaptic inputs with highly stereotypical tuning profiles (**Figure 3C**). Specifically, in all OSGC types the excitatory currents are tuned to the cell’s preferred orientation. Inhibitory currents are tuned to the orthogonal orientation (i.e., 90° angular distance) in mouse horizontal ON OSGCs and all rabbit ON and OFF OSGC types. On the other hand, vertical ON OSGCs and both OFF OSGC types in mouse receive inhibitory inputs that are not tuned to orientation (**Figure 3C**).

How are these tuned inputs generated? In mouse, individually blocking either GABA_A_ or glycine receptors did not produce any significant change in the orientation tuning of excitatory or inhibitory inputs received by ON OSGCs, suggesting that there is a substantial level of redundancy among GABAergic and glycinergic mechanisms (Nath and Schwartz, 2016). Blocking both receptor types simultaneously completely abolished inhibitory currents but, surprisingly, did not affect the tuning of excitatory inputs, which thus must arise through inhibition-independent mechanisms. In rabbit, by contrast, pharmacologically blocking GABA_A_ receptors dramatically reduced the orientation tuning of both excitatory and inhibitory inputs in all OSGC types studied (Venkataramani and Taylor, 2010, 2016). Interestingly, the pharmacological blocks in rabbit revealed cell type-specific differences in how the tuning of synaptic currents is generated (**Table 2**). In particular, OFF vertical OSGCs are normally disinhibited exclusively during preferred orientation stimulation but, upon GABAergic inhibition block, are also disinhibited when orthogonal stimuli are presented. OFF horizontal OSGCs receive strong inhibitory currents exclusively during orthogonal stimulation, but completely lose this inhibition when GABA_A_ receptors are blocked. Finally, ON horizontal OSGCs receive larger inhibitory currents during orthogonal stimulation but, after blocking GABA inhibition, receive inhibitory currents of similar amplitude also in response to preferred stimuli. In all three rabbit OSGC types studied, GABA blockade results in increased excitatory currents received during orthogonal stimulation and, consequently, reduced tuning of excitatory inputs.

These pharmacological experiments in rabbit also uncovered the role played by glycinergic amacrine cells in generating the tuned synaptic inputs received by OSGCs. Even though an early study reported that glycine-mediated inhibition is not strictly required for the orientation-selective firing of rabbit OSGCs (Caldwell et al., 1978), Venkataramani and Taylor (2010, 2016) clearly demonstrated that glycinergic amacrine cells are part of the orientation-selective circuits in the rabbit retina, and that their precise role varies between different orientation-selective circuits. OFF vertical OSGCs, for example, receive direct synaptic inputs from a tonically active glycinergic amacrine cell tuned to orthogonally oriented stimuli. This orientation-selective glycinergic amacrine cell is responsible for the large disinhibition received by OFF vertical OSGCs during preferred stimulation because, upon glycine receptors block, this disinhibition is completely lost (Venkataramani and Taylor, 2010). Also ON horizontal OSGCs seem to receive direct inputs from an orientation-selective glycinergic amacrine cell tuned to orthogonal stimuli (Venkataramani and Taylor, 2016). The authors did not block glycine receptors in the latter study, but could conclude that the tuning of this amacrine cell results from orientation-selective inhibition provided by an upstream GABAergic amacrine cell tuned to preferred orientation, since blocking GABA_A_ receptors does not reduce the amplitude of inhibitory currents received by ON horizontal OSGCs but abolishes the orientation tuning of the currents.

The electrophysiological, pharmacological and ablation experiments described above indicate that, at least in rabbit and zebrafish, synaptic inhibition from amacrine cells plays a crucial role in generating OSGC tuning. In rabbit, orientation-selective inhibition from amacrine cells appears to modulate the tuning of OSGCs, as well as amacrine cells and bipolar cell presynaptic terminals. The pharmacological results in the rabbit retina suggest that, in some cases, orientation-selective amacrine cells provide direct inhibitory inputs to bipolar cell presynaptic terminals (Venkataramani and Taylor, 2010). Whether this is actually the case is still open to investigation. However, some indirect evidence consistent with this idea comes from the larval zebrafish, where the distribution of orientation preferences among orientation-tuned bipolar cell terminals is anti-correlated with that observed in orientation-selective amacrine cells, but correlated with the distribution of OSGCs (Antinucci et al., 2016b).

A recent study in the mouse retina revealed additional heterogeneity among OSGC types (Nath and Schwartz, 2017). The authors showed that mouse OFF OSGCs acquire their tuning by being electrically coupled to orientation-selective amacrine cells. In OFF vertical OSGCs, the coupled amacrine cells have highly elongated, vertically oriented dendritic branches that form electrical synapses with OFF vertical OSGCs through connexin 36-mediated gap junctions. By blocking gap junction-mediated signaling using meclofenamic acid (bath applied) or quinine (intracellular), Nath and Schwartz (2017) demonstrated that these electrical synapses are necessary to produce tuned currents and orientation-selective firing in OFF OSGCs (**Figure 3C** and **Table 2**), therefore uncovering a novel mechanisms underlying orientation selectivity in OSGCs.

**FIGURE 5:**
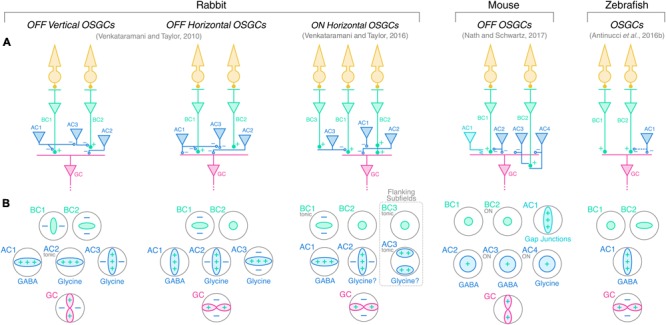
Working models of orientation-selective retinal circuits in vertebrate retinae. **(A)** Proposed circuit diagrams underlying the firing selectivity of different OSGC types in rabbit (left), mouse (middle), and zebrafish (right). Photoreceptors are represented in yellow; bipolar cells (BCs) in green; amacrine cells (ACs) in blue; and ganglion cells (GCs) in red. Cell numbering is used to relate each cell type with the corresponding tuning profile shown in **(B)** below. Excitatory synapses are indicated by ‘+’ symbols (full circles), whereas inhibitory synapses are indicated by ‘–’ (empty circles). Electrical synapses between mouse OFF orientation-selective amacrine cells (AC1) and OFF OSGCs are indicated by the jagged line. Potential but speculative connectivity between AC1 and BC terminals in larval zebrafish is represented by the dashed blue line. **(B)** Response profiles of the cell types represented in A to oriented visual stimuli. ‘+’ and ‘–’ symbols indicate along which axes excitatory (+, green) and inhibitory (–, blue) inputs contribute to the tuning of each cell type. The neurotransmitter identities of the various amacrine cell types are also reported (question marks indicate predicted neurotransmitter identities). GABA, gamma-aminobutyric acid. Tonic inputs are specified in small gray writings. Unless specifically indicated in small gray writings, the response polarity of the various cell types is the same as that of the respective downstream OSGC. In rabbit and zebrafish circuits, orientation selectivity is proposed to originate from orientation-selective GABAergic amacrine cells (AC1), which subsequently generate tuned responses in downstream amacrine, bipolar and/or ganglion cells through inhibitory synapses. In the mouse OFF circuit, AC1 amacrine cells convey orientation selectivity to OFF OSGCs by electrical coupling through gap junctions. Rabbit ON horizontal OSGCs possess two horizontally oriented flanking subfields generated by tonically active amacrine cells (AC3), which are predicted to invert the ON pathway signal by disinhibiting center bipolar cells (BC1) and, therefore, render negative contrast stimuli in the flanking subfields excitatory (dashed box).

The findings presented above allowed researchers to outline working models of the circuit architectures underlying orientation selectivity in the vertebrate retina. Venkataramani and Taylor (2010, 2016), Antinucci et al. (2016b), and Nath and Schwartz (2017) proposed circuit diagrams for rabbit, zebrafish and mouse orientation-selective retinal circuits, respectively. **Figure 5** summarizes their models showing the wiring of bipolar (green) and amacrine (blue) cells upstream of OSGCs (red; **Figure 5A**), as well as their respective tuning profiles in response to oriented visual stimuli (**Figure 5B**). In addition, the neurotransmitter identity of amacrine cell types is also displayed. The whole-cell electrophysiological recordings and pharmacological experiments performed in rabbit and mouse allowed to reveal the synaptic wiring heterogeneity present across different OSGC types (Venkataramani and Taylor, 2010, 2016; Nath and Schwartz, 2017). In contrast, the calcium imaging data in zebrafish did not allow to uncover these details, but outlined the functional connectivity pattern of the orientation-selective retinal circuit in this species (Antinucci et al., 2016b). A unifying feature that appears to be present in all models across the three species is that an orientation-selective amacrine cell type (AC1 in **Figure 5**) is consistently at the origin of orientation selectivity in all tuned neurons of the circuit and, ultimately, OSGCs. Among the orientation-selective amacrine cell types found to date, the most likely candidates for the role of AC1 are: in rabbit, the orientation-biased amacrine cells discovered by Bloomfield (1991, 1994) (**Figure 4A**, right), and the orientation-selective displaced polyaxonal amacrine cells described by Murphy-Baum and Taylor (2015); in mouse, the orientation-selective amacrine cells with vertically oriented dendritic trees reported by Nath and Schwartz (2017); in zebrafish, the two types of orientation-selective amacrine cells found by Antinucci et al. (2016b) (**Figure 4B**). In all these amacrine cell types, orientation tuning seems to be a direct consequence of their highly elongated dendritic fields, and does not appear to be generated by upstream inhibitory mechanisms, as explained above. Thus, their morphological properties make them key cellular substrates underlying the emergence of retinal orientation selectivity. Whether these orientation-selective amacrine cells types found in rabbit, mouse and zebrafish are also present in other vertebrate species is still open to investigation.

## Implications for Visual Processing In Higher Brain Centers

The studies discussed above clearly show that the vertebrate retina is well equipped to generate orientation selectivity. To what extent OSGCs do contribute to orientation-selective firing in higher visual areas is still an open question and a field of intense research. In this context, the classic textbook view that in ‘evolutionary younger’ vertebrates, like mammals, the retinal output largely consists of a simple, pixel-by-pixel representation of the visual scene is challenged. It is now clear that, across vertebrate species, there is a high degree of pre-processing of visual information in the early visual system (Masland and Martin, 2007; Gollisch and Meister, 2010; Niell, 2013). There is no sharp separation between evolutionary older vertebrate visual systems with ‘smart’ retinae producing complex feature selectivity, versus evolutionary younger ones with ‘dull’ retinae performing elementary computations. Even though each vertebrate group exhibits unique characteristics in terms of cell type physiology, morphology, frequency and visual system architecture, it seems that there are fundamental functional properties (with orientation selectivity among them) that are generated very early during visual processing and appear to be almost ubiquitously present across vertebrate retinae. As pointed out before, it is currently unknown how the output of OSGCs is integrated by neurons downstream of the retina. However, recent studies indicate that the representation of orientation-specific information is highly distributed throughout the early visual system, with processing likely occurring at each main level of the visual pathway.

To give some key examples in mammals: cells with high orientation selectivity have been found in the lateral geniculate nucleus (LGN) of mice (Marshel et al., 2012; Piscopo et al., 2013; Scholl et al., 2013; Zhao et al., 2013; Suresh et al., 2016) and monkeys (Smith et al., 1990; Cheong et al., 2013). Orientation bias was reported in cat LGN neurons (Vidyasagar and Urbas, 1982; Soodak et al., 1987; Scholl et al., 2013; Vidyasagar et al., 2015) and, intriguingly, orientation-selective responses were also observed in human LGN using functional magnetic resonance imaging (Ling et al., 2015). In mice, inactivating the visual cortex does not affect orientation tuning in the LGN indicating that cortical feedback is not the source of LGN orientation selectivity (Scholl et al., 2013; Zhao et al., 2013). Intracellular *in vivo* recordings also revealed that mouse orientation-selective LGN neurons receive orientation-tuned excitatory inputs from the retina, pointing toward a retinal contribution from OSGCs (Suresh et al., 2016). But do LGN neurons send tuned inputs to primary visual cortex (V1)? There is now unequivocal evidence that the mouse LGN provides orientation-tuned presynaptic inputs to V1 (Cruz-Martin et al., 2014; Kondo and Ohki, 2016; Sun et al., 2016), and that presynaptic boutons with high orientation selectivity might constitute as much as half of the thalamic inputs in layer 4 (Sun et al., 2016) [but also see (Lien and Scanziani, 2013) and (Kondo and Ohki, 2016)].

Orientation selectivity was also found in the main retinorecipient structure of the mouse brain, the superior colliculus (SC) (Wang et al., 2010; Ahmadlou and Heimel, 2015; Feinberg and Meister, 2015). Like in the LGN, cortical lesions or optogenetic silencing of V1 do not affect the tuning of orientation-selective neurons in the mouse SC, excluding a role for tuned feedback from the cortex (Wang et al., 2010; Ahmadlou and Heimel, 2015). Strikingly, these orientation-selective neurons form columns with similar orientation preference across the depth of the SC superficial layers (Ahmadlou and Heimel, 2015; Feinberg and Meister, 2015). Ahmadlou and Heimel (2015) also showed that orientation preferences of orientation-selective collicular neurons are arranged in a pinwheel-like fashion with the preferred orientation being consistently tangential to the concentric circle around the center of vision. This implies that, unlike the representation of preferred orientations found in V1, not all orientations are equally represented across all visual field locations in the mouse SC.

What could be the advantages of setting up orientation selectivity early in the visual system? In all vertebrates studied, the retina provides a coarse representation of oriented visual elements with 2–4 subpopulations of OSGCs tuned to vertical, horizontal and oblique axes (see **Table 1**). One possibility is that these basic orientations might be used by some downstream cells as building blocks for generating a fine-scale representation in which all orientations are present, like in V1 (Hubel and Wiesel, 1963, 1974; Ohki et al., 2006) and SC (Ahmadlou and Heimel, 2015; Feinberg and Meister, 2015). How fine-scale orientation tuning emerges in V1 is still highly debated, and likely relies on multiple neural mechanisms (Sompolinsky and Shapley, 1997; Ferster and Miller, 2000; Priebe and Ferster, 2012; Vidyasagar and Eysel, 2015; Priebe, 2016). To what extent orientation selectivity in SC and LGN is computed locally or inherited from the retina is currently unknown. However, there is mounting evidence that orientation-selective neurons in the LGN and superior colliculus/optic tectum probably integrate tuned inputs from OSGCs (Hunter et al., 2013; Zhao et al., 2013; Ahmadlou and Heimel, 2015; Suresh et al., 2016). It is reasonable to speculate that retinal orientation selectivity contributes, at least partially, to the encoding of oriented stimuli in higher visual centers. Another potential advantage of having orientation selectivity already in the retina is that orientation-selective retinal outputs can be used by various retinorecipient visual nuclei in parallel. In this way, the same orientation-specific information could subserve multiple functions, such as image formation or gaze control, coordinated by different neural centers.

What experimental strategies could be used to elucidate whether orientation selectivity from the retina plays a role in subsequent stages of visual processing? A first step would be to find the brain targets of OSGC axonal projections (Kim et al., 2008; Huberman et al., 2009; Robles et al., 2014; Martersteck et al., 2017). Also, an important step would be to characterize the functional properties of RGCs labeled by retrograde trans-synaptic circuit tracing from retinorecipient areas that show orientation tuning (Cruz-Martin et al., 2014; Rompani et al., 2017). Another powerful strategy would be to disrupt retinal orientation selectivity through cellular ablations or genetic approaches followed by assessments of orientation tuning in retinal brain targets. This approach has been recently used to dissect the contribution of retinal direction selectivity to motion processing in mouse V1 (Hillier et al., 2017). A molecular marker to label orientation-selective cells in the retina or a genetic strategy to impair retinal orientation selectivity in a targeted manner would greatly help to address these questions. To date, molecular markers associated with retinal orientation-selective cells have been only found in larval zebrafish (Antinucci et al., 2013, 2016b) and mouse (Nath and Schwartz, 2017). In zebrafish, targeted deletion of the gene encoding the transmembrane protein Teneurin-3, expressed in subsets of RGCs and amacrine cells, impairs the development of orientation selectivity in RGCs (Antinucci et al., 2013, 2016b). Moreover, amacrine cells transgenically labeled by a Teneurin-3-specific bacterial artificial chromosome (BAC) show orientation tuning and are required for RGC orientation selectivity (Antinucci et al., 2016b). In mouse, Nath and Schwartz (2017) found that RGCs expressing the adhesion molecule JAM-B (labeled in the JAM-B BAC transgenic line) correspond to the OFF vertically tuned OSGCs. Intriguingly, these cells have been previously shown to be direction-selective in response to small moving spots (Kim et al., 2008). Therefore, this suggests that JAM-B RGCs can be either direction- or orientation-selective depending on the spatial characteristics of the visual stimulus (i.e., moving spots or drifting gratings, respectively). Since JAM-B RGCs send axonal projections to the LGN and SC (Kim et al., 2008), it is reasonable to conclude that OFF vertical OSGCs provide orientation-selective information to these two visual areas. Whether the link between retinal orientation selectivity and these molecular markers (i.e., Teneurin-3 and JAM-B) is conserved across vertebrate species is a matter of ongoing investigations.

## Conclusion

We have explored the different orientation-selective cell types found in vertebrate retinae. We have described their morphological and functional characteristics, as well as the mechanisms underlying their tuned firing. For some orientation-selective cell types, there appear to be strong similarities between different species. Like, for example, the orientation-tuned amacrine cells with highly elongated dendritic arbors observed in rabbit (Bloomfield, 1991, 1994), mouse (Nath and Schwartz, 2017), and zebrafish (Antinucci et al., 2016b) (**Figure 4**). In some cases, homologous cell types in different species have very similar morphological, functional and physiological characteristics, like the ON horizontal OSGCs described in mouse (Nath and Schwartz, 2016) and rabbit (Venkataramani and Taylor, 2016), but show fundamentally different synaptic mechanisms underlying their orientation tuning (**Figure 3** and **Table 2**). This is not surprising given that there are species-specific anatomical features (e.g., eye size) that constrain the way certain computations can be implemented in retinal circuits (Ding et al., 2016; Euler and Baden, 2016).

We also stressed that the contribution of orientation-selective retinal outputs to subsequent stages of visual processing has to be taken into account to understand how orientation selectivity emerges along the vertebrate visual system. Notably, OSGCs constitute a substantial fraction of the whole retinal output. To give some examples, they are ∼15% in mouse (Baden et al., 2016), ∼5–11% in rabbit (Levick, 1967; Venkataramani and Taylor, 2010, 2016), and ∼10% in larval zebrafish (Lowe et al., 2013; Antinucci et al., 2016b). Given the steadily growing interest in retinal orientation selectivity, future studies will surely reveal the precise role OSGCs play in visual processing, as well as the presynaptic cell types and mechanisms in the retina underlying their selective firing. We think that it is crucial to address these questions in different vertebrate species not only to have a better grasp of how neural circuits underlying the same functional task may have evolved, but also to understand how unique anatomical and physiological constrains can influence the implementation of a fundamental neural computation like orientation selectivity in different animals.

## Author Contributions

PA conceived and carried out the literature review research, and wrote the article. RH conceived and revised the literature review and contributed to writing the article.

## Conflict of Interest Statement

The authors declare that the research was conducted in the absence of any commercial or financial relationships that could be construed as a potential conflict of interest.

## References

[B1] Ahmadlou M. Heimel J. A. (2015). Preference for concentric orientations in the mouse superior colliculus. *Nat. Commun.* 6:6773. 10.1038/ncomms7773 25832803 PMC4396361

[B2] Amthor F. R. Takahashi E. S. Oyster C. W. (1989). Morphologies of rabbit retinal ganglion cells with complex receptive fields. *J. Comp. Neurol.* 280 97–121. 10.1002/cne.902800108 2918098

[B3] Antinucci P. Abbas F. Hunter P. R. (2016a). Orientation selectivity in the retina: on cell types and mechanisms. *J. Neurosci.* 36 8064–8066. 10.1523/JNEUROSCI.1527-16.2016 27488626 PMC4971358

[B4] Antinucci P. Nikolaou N. Meyer M. P. Hindges R. (2013). Teneurin-3 specifies morphological and functional connectivity of retinal ganglion cells in the vertebrate visual system. *Cell Rep.* 5 582–592. 10.1016/j.celrep.2013.09.045 24183672 PMC3898612

[B5] Antinucci P. Suleyman O. Monfries C. Hindges R. (2016b). Neural mechanisms generating orientation selectivity in the retina. *Curr. Biol.* 26 1802–1815. 10.1016/j.cub.2016.05.035 27374343 PMC4963213

[B6] Baden T. Berens P. Franke K. Roman Roson M. Bethge M. Euler T. (2016). The functional diversity of retinal ganglion cells in the mouse. *Nature* 529 345–350. 10.1038/nature16468 26735013 PMC4724341

[B7] Bell A. J. Sejnowski T. J. (1997). The “independent components” of natural scenes are edge filters. *Vis. Res.* 37 3327–3338. 10.1016/S0042-6989(97)00121-1 9425547 PMC2882863

[B8] Bloomfield S. A. (1991). Two types of orientation-sensitive responses of amacrine cells in the mammalian retina. *Nature* 350 347–350. 10.1038/350347a0 1706822

[B9] Bloomfield S. A. (1994). Orientation-sensitive amacrine and ganglion cells in the rabbit retina. *J. Neurophysiol.* 71 1672–1691. 10.1152/jn.1994.71.5.1672 8064341

[B10] Brincat S. L. Connor C. E. (2004). Underlying principles of visual shape selectivity in posterior inferotemporal cortex. *Nat. Neurosci.* 7 880–886. 10.1038/nn1278 15235606

[B11] Caldwell J. H. Daw N. W. Wyatt H. J. (1978). Effects of picrotoxin and strychnine on rabbit retinal ganglion cells: lateral interactions for cells with more complex receptive fields. *J. Physiol.* 276 277–298. 10.1113/jphysiol.1978.sp012233 650450 PMC1282424

[B12] Chang L. Tsao D. Y. (2017). The code for facial identity in the primate brain. *Cell* 169 1013–1028.e14. 10.1016/j.cell.2017.05.011 28575666 PMC8088389

[B13] Chen H. Liu X. Tian N. (2014). Subtype-dependent postnatal development of direction- and orientation-selective retinal ganglion cells in mice. *J. Neurophysiol.* 112 2092–2101. 10.1152/jn.00320.2014 25098962 PMC4274919

[B14] Cheong S. K. Tailby C. Solomon S. G. Martin P. R. (2013). Cortical-like receptive fields in the lateral geniculate nucleus of marmoset monkeys. *J. Neurosci.* 33 6864–6876. 10.1523/JNEUROSCI.5208-12.2013 23595745 PMC6618877

[B15] Cruz-Martin A. El-Danaf R. N. Osakada F. Sriram B. Dhande O. S. Nguyen P. L. (2014). A dedicated circuit links direction-selective retinal ganglion cells to the primary visual cortex. *Nature* 507 358–361. 10.1038/nature12989 24572358 PMC4143386

[B16] Dakin S. C. Watt R. J. (2009). Biological ”bar codes” in human faces. *J. Vis.* 9 2.1–10. 10.1167/9.4.2 19757911

[B17] Damjanovic I. Maximova E. Maximov P. Maximov V. (2012). Cardinal difference between the orientation-selective retinal ganglion cells projecting to the fish tectum and the orientation-selective complex cells of the mammalian striate cortex. *J. Integr. Neurosci.* 11 169–182. 10.1142/S0219635212500124 22744823

[B18] Damjanovic I. Maximova E. Maximov V. (2009). On the organization of receptive fields of orientation-selective units recorded in the fish tectum. *J. Integr. Neurosci.* 8 323–344. 10.1142/S0219635209002174 19938209

[B19] Ding H. Smith R. G. Poleg-Polsky A. Diamond J. S. Briggman K. L. (2016). Species-specific wiring for direction selectivity in the mammalian retina. *Nature* 535 105–110. 10.1038/nature18609 27350241 PMC4959608

[B20] Euler T. Baden T. (2016). Computational neuroscience: species-specific motion detectors. *Nature* 535 45–46. 10.1038/nature18454 27350240

[B21] Feinberg E. H. Meister M. (2015). Orientation columns in the mouse superior colliculus. *Nature* 519 229–232. 10.1038/nature14103 25517100

[B22] Ferster D. Miller K. D. (2000). Neural mechanisms of orientation selectivity in the visual cortex. *Annu. Rev. Neurosci.* 23 441–471. 10.1146/annurev.neuro.23.1.441 10845071

[B23] Fisher Y. E. Silies M. Clandinin T. R. (2015). Orientation selectivity sharpens motion detection in drosophila. *Neuron* 88 390–402. 10.1016/j.neuron.2015.09.033 26456048 PMC4664581

[B24] Gollisch T. Meister M. (2010). Eye smarter than scientists believed: neural computations in circuits of the retina. *Neuron* 65 150–164. 10.1016/j.neuron.2009.12.009 20152123 PMC3717333

[B25] Hammond P. (1974). Cat retinal ganglion cells: size and shape of receptive field centres. *J. Physiol.* 242 99–118. 10.1113/jphysiol.1974.sp010696 4436829 PMC1330602

[B26] He S. Levick W. R. Vaney D. I. (1998). Distinguishing direction selectivity from orientation selectivity in the rabbit retina. *Vis. Neurosci.* 15 439–447. 10.1017/S0952523898153038 9685197

[B27] Helmstaedter M. Briggman K. L. Turaga S. C. Jain V. Seung H. S. Denk W. (2013). Connectomic reconstruction of the inner plexiform layer in the mouse retina. *Nature* 500 168–174. 10.1038/nature12346 23925239

[B28] Hillier D. Fiscella M. Drinnenberg A. Trenholm S. Rompani S. B. Raics Z. (2017). Causal evidence for retina-dependent and -independent visual motion computations in mouse cortex. *Nat. Neurosci.* 20 960–968. 10.1038/nn.4566 28530661 PMC5490790

[B29] Hubel D. H. Wiesel T. N. (1962). Receptive fields, binocular interaction and functional architecture in the cat’s visual cortex. *J. Physiol.* 160 106–154. 10.1113/jphysiol.1962.sp006837 14449617 PMC1359523

[B30] Hubel D. H. Wiesel T. N. (1963). Shape and arrangement of columns in cat’s striate cortex. *J. Physiol.* 165 559–568. 10.1113/jphysiol.1963.sp007079 13955384 PMC1359325

[B31] Hubel D. H. Wiesel T. N. (1968). Receptive fields and functional architecture of monkey striate cortex. *J. Physiol.* 195 215–243. 10.1113/jphysiol.1968.sp008455 4966457 PMC1557912

[B32] Hubel D. H. Wiesel T. N. (1974). Sequence regularity and geometry of orientation columns in the monkey striate cortex. *J. Comp. Neurol.* 158 267–293. 10.1002/cne.901580304 4436456

[B33] Huberman A. D. Wei W. Elstrott J. Stafford B. K. Feller M. B. Barres B. A. (2009). Genetic identification of an on-off direction-selective retinal ganglion cell subtype reveals a layer-specific subcortical map of posterior motion. *Neuron* 62 327–334. 10.1016/j.neuron.2009.04.014 19447089 PMC3140054

[B34] Hunter P. R. Lowe A. S. Thompson I. D. Meyer M. P. (2013). Emergent properties of the optic tectum revealed by population analysis of direction and orientation selectivity. *J. Neurosci.* 33 13940–13945. 10.1523/JNEUROSCI.1493-13.2013 23986231 PMC3756745

[B35] Johnston J. Ding H. Seibel S. H. Esposti F. Lagnado L. (2014). Rapid mapping of visual receptive fields by filtered back projection: application to multi-neuronal electrophysiology and imaging. *J. Physiol.* 592 4839–4854. 10.1113/jphysiol.2014.276642 25172952 PMC4259530

[B36] Johnston J. Lagnado L. (2015). General features of the retinal connectome determine the computation of motion anticipation. *Elife* 4:e06250. 10.7554/eLife.06250 25786068 PMC4391023

[B37] Kim I. J. Zhang Y. Yamagata M. Meister M. Sanes J. R. (2008). Molecular identification of a retinal cell type that responds to upward motion. *Nature* 452 478–482. 10.1038/nature06739 18368118

[B38] Kondo S. Ohki K. (2016). Laminar differences in the orientation selectivity of geniculate afferents in mouse primary visual cortex. *Nat. Neurosci.* 19 316–319. 10.1038/nn.4215 26691830

[B39] Krizhevsky A. Sutskever I. Hinton G. E. (2012). “Imagenet classification with deep convolutional neural networks,” in *Paper Presented at the Advances in Neural Information Processing systems* Lake Tahoe, NV.

[B40] LeCun Y. Bengio Y. Hinton G. (2015). Deep learning. *Nature* 521 436–444. 10.1038/nature14539 26017442

[B41] Leventhal A. G. Schall J. D. (1983). Structural basis of orientation sensitivity of cat retinal ganglion cells. *J. Comp. Neurol.* 220 465–475. 10.1002/cne.902200408 6643739

[B42] Levick W. R. (1967). Receptive fields and trigger features of ganglion cells in the visual streak of the rabbits retina. *J. Physiol.* 188 285–307. 10.1113/jphysiol.1967.sp008140 6032202 PMC1396015

[B43] Levick W. R. Thibos L. N. (1980). Orientation bias of cat retinal ganglion cells. *Nature* 286 389–390. 10.1038/286389a0 7402319

[B44] Levick W. R. Thibos L. N. (1982). Analysis of orientation bias in cat retina. *J. Physiol.* 329 243–261. 10.1113/jphysiol.1982.sp014301 7143249 PMC1224778

[B45] Lien A. D. Scanziani M. (2013). Tuned thalamic excitation is amplified by visual cortical circuits. *Nat. Neurosci.* 16 1315–1323. 10.1038/nn.3488 23933748 PMC3774518

[B46] Ling S. Pratte M. S. Tong F. (2015). Attention alters orientation processing in the human lateral geniculate nucleus. *Nat. Neurosci.* 18 496–498. 10.1038/nn.3967 25730671 PMC4556110

[B47] Lowe A. S. Nikolaou N. Hunter P. R. Thompson I. D. Meyer M. P. (2013). A systems-based dissection of retinal inputs to the zebrafish tectum reveals different rules for different functional classes during development. *J. Neurosci.* 33 13946–13956. 10.1523/JNEUROSCI.1866-13.2013 23986232 PMC3756746

[B48] Marshel J. H. Kaye A. P. Nauhaus I. Callaway E. M. (2012). Anterior-posterior direction opponency in the superficial mouse lateral geniculate nucleus. *Neuron* 76 713–720. 10.1016/j.neuron.2012.09.021 23177957 PMC3517882

[B49] Martersteck E. M. Hirokawa K. E. Evarts M. Bernard A. Duan X. Li Y. (2017). Diverse central projection patterns of retinal ganglion cells. *Cell Rep.* 18 2058–2072. 10.1016/j.celrep.2017.01.075 28228269 PMC5357325

[B50] Masland R. H. Martin P. R. (2007). The unsolved mystery of vision. *Curr. Biol.* 17 R577–R582. 10.1016/j.cub.2007.05.040 17686423

[B51] Maturana H. R. Frenk S. (1963). Directional movement and horizontal edge detectors in the pigeon retina. *Science* 142 977–979. 10.1126/science.142.3594.977 14069232

[B52] Mazurek M. Kager M. Van Hooser S. D. (2014). Robust quantification of orientation selectivity and direction selectivity. *Front. Neural Circuits* 8:92. 10.3389/fncir.2014.00092 25147504 PMC4123790

[B53] Murphy-Baum B. L. Taylor W. R. (2015). The synaptic and morphological basis of orientation selectivity in a polyaxonal amacrine cell of the rabbit retina. *J. Neurosci.* 35 13336–13350. 10.1523/JNEUROSCI.1712-15.2015 26424882 PMC4588608

[B54] Nath A. Schwartz G. W. (2016). Cardinal orientation selectivity is represented by two distinct ganglion cell types in mouse retina. *J. Neurosci.* 36 3208–3221. 10.1523/JNEUROSCI.4554-15.2016 26985031 PMC4792935

[B55] Nath A. Schwartz G. W. (2017). Electrical synapses convey orientation selectivity in the mouse retina. *Nat. Commun.* 8:2025. 10.1038/s41467-017-01980-9 29229967 PMC5725423

[B56] Niell C. M. (2013). Vision: more than expected in the early visual system. *Curr. Biol.* 23 R681–R684. 10.1016/j.cub.2013.07.049 23968921

[B57] Niell C. M. Stryker M. P. (2008). Highly selective receptive fields in mouse visual cortex. *J. Neurosci.* 28 7520–7536. 10.1523/JNEUROSCI.0623-08.2008 18650330 PMC3040721

[B58] Nikolaou N. Lowe A. S. Walker A. S. Abbas F. Hunter P. R. Thompson I. D. (2012). Parametric functional maps of visual inputs to the tectum. *Neuron* 76 317–324. 10.1016/j.neuron.2012.08.040 23083735 PMC4516722

[B59] Ohki K. Chung S. Kara P. Hubener M. Bonhoeffer T. Reid R. C. (2006). Highly ordered arrangement of single neurons in orientation pinwheels. *Nature* 442 925–928. 10.1038/nature05019 16906137

[B60] Olshausen B. A. Field D. J. (1996). Emergence of simple-cell receptive field properties by learning a sparse code for natural images. *Nature* 381 607–609. 10.1038/381607a0 8637596

[B61] Passaglia C. L. Troy J. B. Ruttiger L. Lee B. B. (2002). Orientation sensitivity of ganglion cells in primate retina. *Vis. Res.* 42 683–694. 10.1016/S0042-6989(01)00312-1 11888534 PMC6880403

[B62] Pearson J. T. Kerschensteiner D. (2015). Ambient illumination switches contrast preference of specific retinal processing streams. *J. Neurophysiol.* 114 540–550. 10.1152/jn.00360.2015 25995351 PMC4509391

[B63] Peichl L. Wassle H. (1983). The structural correlate of the receptive field centre of alpha ganglion cells in the cat retina. *J. Physiol.* 341 309–324. 10.1113/jphysiol.1983.sp014807 6620182 PMC1195336

[B64] Piscopo D. M. El-Danaf R. N. Huberman A. D. Niell C. M. (2013). Diverse visual features encoded in mouse lateral geniculate nucleus. *J. Neurosci.* 33 4642–4656. 10.1523/JNEUROSCI.5187-12.2013 23486939 PMC3665609

[B65] Priebe N. J. (2016). Mechanisms of orientation selectivity in the primary visual cortex. *Annu. Rev. Vis. Sci.* 2 85–107. 10.1146/annurev-vision-111815-114456 28532362

[B66] Priebe N. J. Ferster D. (2012). Mechanisms of neuronal computation in mammalian visual cortex. *Neuron* 75 194–208. 10.1016/j.neuron.2012.06.011 22841306 PMC3477598

[B67] Robles E. Laurell E. Baier H. (2014). The retinal projectome reveals brain-area-specific visual representations generated by ganglion cell diversity. *Curr. Biol.* 24 2085–2096. 10.1016/j.cub.2014.07.080 25155513

[B68] Rompani S. B. Mullner F. E. Wanner A. Zhang C. Roth C. N. Yonehara K. (2017). Different modes of visual integration in the lateral geniculate nucleus revealed by single-cell-initiated transsynaptic tracing. *Neuron* 93 767–776.e6. 10.1016/j.neuron.2017.01.028 28231464 PMC5330803

[B69] Sanes J. R. Masland R. H. (2015). The types of retinal ganglion cells: current status and implications for neuronal classification. *Annu. Rev. Neurosci.* 38 221–246. 10.1146/annurev-neuro-071714-034120 25897874

[B70] Scholl B. Tan A. Y. Corey J. Priebe N. J. (2013). Emergence of orientation selectivity in the mammalian visual pathway. *J. Neurosci.* 33 10616–10624. 10.1523/JNEUROSCI.0404-13.2013 23804085 PMC3693051

[B71] Sernagor E. Grzywacz N. M. (1995). Emergence of complex receptive field properties of ganglion cells in the developing turtle retina. *J. Neurophysiol.* 73 1355–1364. 10.1152/jn.1995.73.4.1355 7643153

[B72] Shou T. Leventhal A. G. Thompson K. G. Zhou Y. (1995). Direction biases of x and y type retinal ganglion cells in the cat. *J. Neurophysiol.* 73 1414–1421. 10.1152/jn.1995.73.4.1414 7643156

[B73] Simoncelli E. P. Olshausen B. A. (2001). Natural image statistics and neural representation. *Annu. Rev. Neurosci.* 24 1193–1216. 10.1146/annurev.neuro.24.1.1193 11520932

[B74] Smith E. L. III Chino Y. M. Ridder W. H. III Kitagawa K. Langston A. (1990). Orientation bias of neurons in the lateral geniculate nucleus of macaque monkeys. *Vis. Neurosci.* 5 525–545. 10.1017/S0952523800000699 2085469

[B75] Sompolinsky H. Shapley R. (1997). New perspectives on the mechanisms for orientation selectivity. *Curr. Opin. Neurobiol.* 7 514–522. 10.1016/S0959-4388(97)80031-1 9287203

[B76] Soodak R. E. Shapley R. M. Kaplan E. (1987). Linear mechanism of orientation tuning in the retina and lateral geniculate nucleus of the cat. *J. Neurophysiol.* 58 267–275. 10.1152/jn.1987.58.2.267 3655866

[B77] Sun W. Tan Z. Mensh B. D. Ji N. (2016). Thalamus provides layer 4 of primary visual cortex with orientation- and direction-tuned inputs. *Nat. Neurosci.* 19 308–315. 10.1038/nn.4196 26691829 PMC4731241

[B78] Suresh V. Ciftcioglu U. M. Wang X. Lala B. M. Ding K. R. Smith W. A. (2016). Synaptic contributions to receptive field structure and response properties in the rodent lateral geniculate nucleus of the thalamus. *J. Neurosci.* 36 10949–10963. 10.1523/JNEUROSCI.1045-16.2016 27798177 PMC5098835

[B79] Thibos L. N. Levick W. R. (1985). Orientation bias of brisk-transient y-cells of the cat retina for drifting and alternating gratings. *Exp. Brain Res.* 58 1–10. 10.1007/BF00238948 3987841

[B80] Venkataramani S. Taylor W. R. (2010). Orientation selectivity in rabbit retinal ganglion cells is mediated by presynaptic inhibition. *J. Neurosci.* 30 15664–15676. 10.1523/JNEUROSCI.2081-10.2010 21084622 PMC3135107

[B81] Venkataramani S. Taylor W. R. (2016). Synaptic mechanisms generating orientation selectivity in the on pathway of the rabbit retina. *J. Neurosci.* 36 3336–3349. 10.1523/JNEUROSCI.1432-15.2016 26985041 PMC4792943

[B82] Vidyasagar T. R. Eysel U. T. (2015). Origins of feature selectivities and maps in the mammalian primary visual cortex. *Trends Neurosci.* 38 475–485. 10.1016/j.tins.2015.06.003 26209463

[B83] Vidyasagar T. R. Jayakumar J. Lloyd E. Levichkina E. V. (2015). Subcortical orientation biases explain orientation selectivity of visual cortical cells. *Physiol. Rep.* 3:e12374. 10.14814/phy2.12374 25855249 PMC4425978

[B84] Vidyasagar T. R. Urbas J. V. (1982). Orientation sensitivity of cat lgn neurones with and without inputs from visual cortical areas 17 and 18. *Exp. Brain Res.* 46 157–169. 10.1007/BF00237172 7095028

[B85] Wang L. Sarnaik R. Rangarajan K. Liu X. Cang J. (2010). Visual receptive field properties of neurons in the superficial superior colliculus of the mouse. *J. Neurosci.* 30 16573–16584. 10.1523/JNEUROSCI.3305-10.2010 21147997 PMC3073584

[B86] Zhao X. Chen H. Liu X. Cang J. (2013). Orientation-selective responses in the mouse lateral geniculate nucleus. *J. Neurosci.* 33 12751–12763. 10.1523/JNEUROSCI.0095-13.2013 23904611 PMC3728687

